# What is important in evaluating health care quality? An international comparison of user views

**DOI:** 10.1186/1472-6963-5-16

**Published:** 2005-02-21

**Authors:** Peter P Groenewegen, Jan J Kerssens, Herman J Sixma, Ingrid van der Eijk, Wienke GW Boerma

**Affiliations:** 1 NIVEL, Netherlands Institute for Health Services Research, PO Box 1568, 3500 BN, The Netherlands; 2 At the time of this research: department of Gastroenterology & Hepatology, University Hospital Maastricht, PO Box 5800, 6202 AZ Maastricht, The Netherlands

## Abstract

**Background:**

Quality of care from the perspective of users is increasingly used in evaluating health care performance. Going beyond satisfaction studies, quality of care from the users' perspective is conceptualised in two dimensions: the importance users attach to aspects of care and their actual experience with these aspects. It is well established that health care systems differ in performance. The question in this article is whether there are also differences in what people in different health care systems view as important aspects of health care quality. The aim is to describe and explain international differences in the importance that health care users attach to different aspects of health care.

**Methods:**

Data were used from different studies that all used a version of the QUOTE-questionnaire that measures user views of health care quality in two dimensions: the importance that users attach to aspects of care and their actual experience. Data from 12 European countries and 5133 individuals were used. They were analysed using multi-level analysis.

**Results:**

Although most of the variations in importance people attach to aspects of health care is located at the individual level, there are also differences between countries. The ranking of aspects shows similarities. 'My GP should always take me seriously' was in nearly all countries ranked first, while an item about waiting time in the GP's office was always ranked lowest.

**Conclusion:**

Differences between countries in how health care users value different aspects of care are difficult to explain. Further theorising should take into account that importance and performance ratings are positively related, that people compare their experiences with those of others, and that general and instrumental values might be related through the institutions of the health care system.

## Background

Large differences between countries exist in the use, costs, quality, and accessibility of health services [1]. Also large differences exist between countries in health care performance and in people's evaluations of their health care system [2-7]. But do these differences also exist in what people from different countries view as important in evaluating health care quality? The World Health Report 2000 has been criticized on its assumption of a universal value base to all health care systems; values such as responsiveness may be valued differently in different countries [8]. In this article we address this issue by comparing what people find important in general practice care in different countries. Grol et al studied patients' priorities in general practice [9]. They found both many similarities and differences between countries. Particularly, doctor-patient communication and accessibility of services were common priorities among general practice visitors in different countries. Service aspects, such as waiting times, were considered less important.

In this study we did a secondary analysis on surveys of patient views on quality of health care. Patients' views were measured using the QUOTE-questionnaire – with the acronym QUOTE standing for **QU**ality **O**f care **T**hrough the patients' **E**yes – that distinguishes two quality of care dimensions: performance and importance [10]. Performance relates to the actual experience of the use of health care services (rather than a patient satisfaction judgement), which is in line with recent developments within health services research. Importance refers to the fact that people see some features of health services as more significant than others. They reflect what people see as desired qualities in health care. This approach avoids problems with conceptualising people's evaluations of health care in terms of satisfaction (usually high levels of satisfaction, not specific enough to be used in quality improvement) and expectations (ambiguous relations between expectations and actual experiences).

To select relevant quality of care aspects, a general and a disease-specific approach was followed, using focus group discussions [10]. With this procedure a series of QUOTE-questionnaires has been tailored to the needs of various patient groups. In these QUOTE-questionnaires the expectations of people are reflected in the statements included in the instruments. These questionnaires have been used in several studies in different countries.

### Research questions

In this article we first compare the importance dimension of QUOTE across several European countries and Israel to gain insight in the similarities and differences in people's views on quality of care. Secondly, we will look at the relationship between importance and performance ratings as part of an explorative analysis to explain differences in importance ratings between patients and/or countries. The general research question is:

Do patients in different European countries think differently about the relative importance of various aspects of quality of care, and if so, how can these differences be explained?

This general question is divided into the following separate questions:

a. To what extent do the importance judgements of patients cluster within countries when individual characteristics of patients are taken into account?

b. Does the ranking of importance judgements vary between countries?

c. What is the relationship between the average performance of health care systems, as judged by patients, and the individual importance judgements?

When we compare the importance judgements of patients between countries, we will take into account individual characteristics of respondents to rule out differences in the composition of the groups of respondents. With respect to the relationship between importance and performances scores it might be anticipated that in general people attach more importance to those aspects that they actually experience less often. Analogous to the economic mechanism of decreasing marginal utility, e.g. quick service without waiting time in the doctor's office might be valued as less important, if in general services are quick and people don't have to wait long. However, at the same time it can be hypothesized that it's no use aspiring to something that nobody has. If quality of care ratings, as seen through the eyes of the patient, are low on the average and if there is small variation in these performance ratings between individuals, people will probably not find these aspects important. This idea is based on a mechanism of social comparison [11].

## Methods

### Material

The First Dutch QUOTE-questionnaires (for disabled persons, COPD, arthritis and frail elderly people) served as a starting point for our database [10,12]. These questionnaires contain 16 general importance and performance items. In the SCOPE-project (Supporting Clinical Outcomes in Primary Care for the Elderly) the QUOTE-elderly was used in Finland, Ireland and the Netherlands [13]. A large contribution to the database comes from an international study of patients with inflammatory bowel disease (IBD) in eight countries [14]. This study used ten generic questions (of the original 16) relating to GPs. Additional material was obtained from the UK (QUOTE-disabled) and from Belarus and the Ukraine [15,16].

The QUOTE-questionnaires have been translated in the context of different projects. In all but two cases backward-forward translations have been used. Answering formats of importance items were: Not important (1); Fairly important (2); Important (3); and Extremely important (4). The answering formats for the performance items were: No (1); Not really (2); On the whole, yes (3); and Yes (4). The equivalence of the answering formats in different languages has not been assessed. The wording of the importance items that were used in the QUOTE-questionnaires are presented in tables 2 and 5 and throughout the result section of the article. The performance items ask for the actual experience of respondents. One of the importance items is, e.g., 'my GP should always take me seriously'. The corresponding performance item is: my GP always takes me seriously'. QUOTE-items included in the analysis refer to the organisation of health care services and the care giving process.

**Table 1:** Number of respondents in health care user groups and countries

Country	User group	Selection of users	N per user group	N per country
Belarus	GP patients	GP office	500	500
Denmark	IBD^a^	hospital files	102	102
Finland	Elderly	PHC files	143	143
Greece	IBD	hospital files	96	96
Ireland	IBD	hospital files	57	73
	Elderly	Homecare Organisation files	16	
Israel	IBD	hospital files	46	46
Italy	IBD	hospital files	201	201
Netherlands	Migrants	Snowball method	152	2873
	IBD	hospital files	192	
	Elderly	GP files	338	
	Disabled	GP files/patient organisation	334	
	Diabetes	GP files/patient organisation	681	
	COPD	GP files/patient organisation	604	
	Arthritis	GP files/patient organisation	572	
Norway	IBD	hospital files	93	93
Portugal	IBD	hospital files	36	36
UK	Disabled	GP files	480	480
Ukraine	GP patients	GP office	490	490
				
Total			5133	5133

^a ^IBD inflammatory bowel disease

**Table 2:** Descriptive statistics for importance items: mean, variance at user level, variance at country level, intra-class correlation coefficient, uncorrected (ICCu) and corrected for age and sex (ICCc)

**Item**	**My GP should**	**Mean**	**Var Users**	**Var Country**	**ICCu**	**ICCc**
3	always take me seriously	3.45	.377	.044	.105	.092
6	inform me, in understandable language, about the medicines that are prescribed for me	3.35	.532	.059	.100	.107
1	have a good understanding of my problems	3.24	.615	.147	.193	.087
9	make sure that I can see a specialist within 2 weeks after being referred to him/her	3.13	.591	.198	.251	.256
4	always keep appointments punctually	3.10	.545	.074	.120	.113
2	allow me to have an input into the decisions regarding the treatment or help I receive	3.07	.648	.071	.099	.063
7	prescribe medicines which are fully covered by the National Health System or social services	3.05	.896	.100	.100	.113
8	always be easy to reach by telephone	3.02	.509	.060	.092	.111
10	always communicate with other health and social care providers about the services I require	2.90	.650	.084	.114	.116
5	not keep me in the waiting room for more than 15 minutes	2.54	1.016	.062	.058	.087

**Table 3:** Mean scores of ten importance items per country

	1*	2*	3*	4*	5*	6*	7*	8*	9*	10*
Belarus**	2.42	2.61	3.21	3.34	2.59	3.04		2.98		2.68
Denmark	3.23	3.22	3.47	2.75	2.37	3.57	2.43	2.62	3.10	2.57
Finland**		2.91	3.03	2.84		2.87		2.86		3.00
Greece	3.46	3.10	3.51	3.25	2.64	3.34	3.09	3.23	3.03	2.93
Ireland	3.47	3.15	3.60	2.90	2.42	3.55	3.02	3.01	3.21	2.96
Israel	3.71	3.70	3.84	3.52	3.07	3.75	3.40	3.14	3.51	3.12
Italy	3.10	2.89	3.23	2.63	1.95	3.12	2.79	2.70	2.03	2.39
Netherlands	3.21	3.28	3.59	3.24	2.50	3.42	3.01	3.33	3.27	3.29
Norway	3.42	3.14	3.60	3.08	2.46	3.57	3.00	2.81	3.17	2.56
Portugal	3.61	2.94	3.61	3.19	2.78	3.42	3.64	3.42	3.44	3.28
UK	3.37	3.20	3.44	3.02	2.57	3.40	3.21	3.27	3.46	3.20
Ukraine**	2.68	2.77	3.37	3.46	2.71	3.21		2.88		2.92
Overall	3.24	3.07	3.45	3.10	2.54	3.35	3.05	3.02	3.13	2.90

* See table 2 for the wording of the items.

** Not all ten items were used in these countries.

**Table 4:** Ranking of items by mean importance within each country; r1-r10 is the ranking, cell entries contain the item number corresponding to that rank.

Country	r1	r2	r3	r4	r5	r6	r7	r8	r9	r10
Denmark	6	3	1	2	9	4	8	10	7	5
Greece	3	1	6	4	8	2	7	9	10	5
Ireland	3	6	1	9	2	7	8	10	4	5
Israel	3	6	1	2	4	9	7	8	10	5
Italy	3	6	1	2	7	8	4	10	9	5
Netherlands	3	6	8	10	2	9	4	1	7	5
Norway	3	6	1	9	2	4	7	8	10	5
Portugal	7	1	3	9	6	8	10	4	2	5
UK	9	3	6	1	8	7	10	2	4	5

**Table 5:** Pearson correlation coefficients between importance- and performance items on individual level and between average performance items in each country and the individual importance scores (mixed level)

Importance/performance items	Individual level	Mixed level
have a good understanding of my problems	.346 **	.322
allow me to have an input into the decisions regarding the treatment or help I receive	.277 **	.263
always take me seriously	.126 **	.167
always keep appointments punctually	.140 **	.081
not keep me in the waiting room for more than 15 minutes	.150 **	.054
inform me, in understandable language, about the medicines that are prescribed for me	.179 **	.170
prescribe medicines which are fully covered by the National Health System or social services	.080 **	-.057
always be easy to reach by telephone	.163 **	.184
make sure that I can see a specialist within 2 weeks after being referred to him/her	.135 **	.119
always communicate with other health and social care providers about the services I require	.281 **	.287

** p < .01

Table 1 gives the number of respondents in each user group and country and the selection of respondents. In the case of Belarus and Ukraine respondents were selected by distributing questionnaires to people who visited general practices. Response rates are not available for these two countries. In all other countries but one addresses were randomly selected from the files of health care (and in one case home care) institutions and (in The Netherlands) from membership lists of voluntary patient organisations, irrespective of actual visits to a GP. In the case of the QUOTE-Migrants, respondents from ethnic groups in the Netherlands were selected using a snowball sampling method; data for these respondents were collected through oral interviews in the respondents' mother language. In all other cases postal questionnaires were used, followed by one or two reminders. Response rates vary between 35% (elderly in the Netherlands) and 78% (the average of the IBD samples).

### Statistical analysis

All 5133 health care users reported for each of (maximum) ten items their importance and performance ratings. The importance ratings are dependent variables in a series of statistical analyses with patients hierarchically nested in countries. In contrast to traditional forms of analysis of variance in which factors have 'fixed' effects, countries are considered to have 'random' effects. Such a variance component model is preferred over traditional analysis if the number of categories exceeds ten [17,18]. The degree of resemblance between patients belonging to the same country can be expressed by the intraclass correlation coefficient (ICC). If there is no resemblance between patients within countries, the ICC is zero or near zero. An ICC of .15 is considered quite high [19]. Most commonly ICCs are lower. For instance, the median ICC calculated for more that 1000 primary care variables, was .01 [20].

The ICC is statistically defined as the variance between countries divided by the total variance. An ICC of zero therefore implies no variance between countries, indicating the absence of differences between countries in patients' importance ratings. Age and sex were included as covariates to take into account differences in the composition of responder groups in the different countries, because of their association with importance scores [9,10,21-23]. Correction for different user groups turned out to be impossible due to the small number of countries for some user groups. Differences in number of cases between countries were taken into account in the statistical analysis. The estimates of country parameters are more precise with larger numbers per country.

In order to explore the relationship between importance and performance ratings Pearson correlations were calculated between the performance items, both at the individual level and aggregated to country level, and the importance rating on user level. In the introduction we have put forward two hypothetical relations between variation of performance within the countries and the importance ratings. To look at the relation between variation of performance and importance ratings, a distinction was made between countries with large variation in experienced health care quality and countries with smaller variation. Based on the mean standard deviation (SD) of all ten performance items, the countries were equally split into: Denmark, Italy, Belarus, Ireland, Ukraine and Portugal (mean SDs ranging from 1.17 to 1.40) indicating countries with high variation and Greece, Finland, Israel, Norway, Netherlands and UK (mean SDs ranging from .82 to 1.08) indicating countries with low variation. Furthermore, explore the relationship between variation in performance at country level, individual respondents performance experience and their importance ratings, we have divided the individual respondents into those who experienced high performance and those who experienced low performance. To keep these distinctions conveniently arranged in one table, we recoded the individual performance scores from the four point scale to a two point scale, with the combination of 'No' and 'No, not really', indicating poor quality, versus 'On the whole, yes' and 'Yes'.

## Results

We start the presentation of the results with a description of the overall importance that respondents in all twelve countries attached to the different aspects and the clustering of their answers within countries. We then move to the differences in the ranking of aspects between countries. Finally, we will present results for the relationship between the actual experiences of respondents, both individually and aggregated to an average for each country, and the importance they attach to the different aspects.

### Importance judgements and clustering

As shown in table 2, 'The GP should always take me seriously' is seen as most important, halfway between 'important' and 'extremely important' on the Likert scale. Less than 1% of users rated this item as 'not important' (not in table). The differences between users as well as countries for this aspect are the smallest of all aspects (smallest variance, both on user- and country level). 'The GP should not keep me in the waiting room for more than 15 minutes' is seen as least important, halfway between 'important' and 'fairly important'. About 20% of users rated this aspect as 'not important' (not in table). The differences between users for this aspect are largest (highest variance on user level). The importance of 'The GP should make sure that I can see a specialist within 2 weeks' shows the biggest differences between countries. The uncorrected ICCs vary from low (.058 aspect 5) to high (.251 aspect 9). The sex-age adjusted ICCs are a little (7%) lower on average, but still range to high.

In order to explore differences between user groups, we have computed intra-class correlation coefficients for user groups within the Netherlands only. These coefficients are on average higher than those regarding countries. We also analysed the differences between countries within the IBD patient groups. These differences are much like the figures of table 2. So the estimated intra-class coefficients of table 2 seem to refer more to differences between countries than differences between user groups.

### Differences between countries and ranking

The variation between the countries for each aspect is illustrated in table 3 by mean importance scores for all aspects. For instance, 'The GP should always take me seriously' is seen as most important in Israel and as least important in Finland. 'The GP should not keep me in the waiting room for more than 15 minutes' also is seen as most important in Israel but as least important in Italy.

In order to look at the consistency of user views across the different countries, the ten importance aspects were ranked according to their mean value within each country. Table 4 gives the ranks for countries where all ten aspects are available. Some rankings differ between countries. For instance in Denmark 'My GP should inform me, in understandable language, about the medicines that are prescribed for me' is ranked first. In Portugal it is 'My GP should prescribe medicines which are fully covered by the National Health System or social services'. But there is also a general pattern. The service aspect 'My GP should not keep me in the waiting room for more than 15 minutes' is ranked last in all countries, while 'My GP should always take me seriously' is ranked high in all countries.

### Importance and performance

Looking at the relationship between importance and performance ratings by means of correlation coefficients, table 5 shows that the correlation coefficients on individual level are all positive and range from small (.080) to moderate (.346). Because of the large number of respondents, even the small correlations are statistically significant. The correlation between the average performance in each country and the individual importance vary also from small (.054) to moderate (.322). Because of the small number of countries these correlation coefficients are not significantly different from zero. Except for one aspect 'My GP should prescribe medicines which are fully covered by the National Health System or social services' all correlations are positive, while we anticipated negative correlations.

If we take into account the variation in performance ratings within countries, the positive relationship between importance and performance ratings is somewhat stronger in the 'high variation' mode compared to the 'low variation' mode. This can be seen by comparing the difference between the first two columns of table 6 and those between the last two columns. We had specifically expected to find a difference between the last two columns of table 6, i.e. within countries with low variation in aggregate performance. However, for only half of the items the difference is statistically significant.

**Table 6:** Mean scores of ten importance items according to variation in performance items per country and individual scores on corresponding performance items (Low vs High performance)

	Country level:	High variation in performance		Low variation in performance	
		
	Individual level:	Low performance	High performance	Low performance	High performance
1	should have a good understanding of my problems	2.06	2.92	*3.04*	3.28
2	should allow me to have an input into the decisions regarding the treatment or help I receive	2.27	3.03	*3.11*	3.30
3	should always take me seriously	2.99	3.35	3.66	3.55
4	should always keep appointments punctually	3.01	3.32	3.08	3.22
5	should not keep me in the waiting room for more than 15 minutes	2.26	2.82	*2.42*	2.60
6	should inform me, in understandable language, about the medicines that are prescribed for me	2.80	3.24	*3.28*	3.43
7	should prescribe medicines which are fully covered by the National Health System or social services	2.74	2.95	2.98	3.09
8	should always be easy to reach by telephone	2.63	3.04	3.29	3.34
9	should make sure that I can see a specialist within 2 weeks after being referred to him/her	2.29	2.67	3.26	3.34
10	should always communicate with other health and social care providers about the services I require	2.42	2.98	*3.12*	3.30

Italic means in column Low variation at country level/Low performance at individual level differ statistically significant from means in column Low variation at country level/High performance at individual level (Scheffé contrasts).

Overall, importance scores are somewhat lower in countries with high variation in performance ratings as compared to countries with low variation in performance scores.

## Discussion

The objective of our study was to gain insight into similarities and differences in the value users of health care in different countries attach to aspects of care. As an indicator of these values, importance scores of the QUOTE-questionnaires were used. These scores reflect what is important in evaluating health care quality according to users. Our results show that health care users in different countries to some extent think differently about the relative importance of various aspects of quality of care. Intra-class correlation coefficients were calculated to measure the difference between countries. They range from low to high. Sex-age adjusted intra-class correlation coefficients were only slightly lower. This means that demographic differences between the groups that filled in the questionnaires in different countries cannot explain the differences in average importance between the countries.

Although there are differences between countries, the importance rankings of the aspects also show consensus. 'My GP should always take me seriously' is nearly always ranked highest, while the item about waiting time is always ranked as least important. Since we only analysed a small sample of countries, it is difficult to generalise this result. However, it might say something about a hierarchy of these instrumental health care values, suggesting that values concerning respectfulness are seen as more important than service aspects, such as waiting time. This is in line with the findings of Grol et al. [9]. There are no accepted explanations for these value differences between countries. General theories about dimensions of culture see culture as the independent variable, explaining differences between countries in institutions and organisations [24,25]. A more specific application in the health care field is Payer's book Medicine and Culture that relates differences in culture to variations in the practice of medicine [26]. In this article, however, differences in values is what we want to explain.

In general, there is a positive relation between what people find important and their experiences, both on an individual level and related to the average experience in a country. The positive relation between importance and experience could probably be explained by a general tendency of cognitive consistency [27] or alternatively by processes of selection where people try to find those health care providers that do what they value most. This alternative hypothesis would mean that the correlation between importance and experience is stronger the more freedom of choice of health care provider people have. One of the assumptions behind the QUOTE-questionnaires is that importance and experience are two different aspects, together constituting quality judgements. The correlation at the individual level is not so high as to invalidate this assumption.

By looking at the variation in the performance scores, we have tried to include social comparison mechanisms into the analysis. It can be argued that if people's individual experience indicates low performance for certain aspects in countries, they will value these aspects as more important. However, such a hypothesis has to be rejected on the basis of the results presented in this article. A contrasting hypothesis, that in countries with low variation in actual experiences, people who experience low performance themselves, will not aspire to something that is apparently (from their own and others' experience) out of reach is only partly supported by our findings. Although people in this low variation condition have significantly lower importance scores for half of the items, still the differences in the high variation condition are higher.

Looking at the individual aspects, it can be argued that people don't find issues important if they are more or less guaranteed by the health care system. An example for this is the issue of prescribing pharmaceuticals that are fully covered by the health care insurance plans of patients. On the basis of the material presented in this article this hypothesis too has to be rejected. People in countries with low levels of cost sharing in this field, found this item more important than people in countries with higher levels of cost sharing. However, an alternative explanation for this finding could be that structural aspects of the health care system, e.g. on the dimension public – private, reflect general values [28,29]. If these general values also relate to instrumental values, than the relationship we found is understandable: the people in countries that took the pain to organise their health care system in a way that financial access is very good, might find this issue more important. In this explanation the mechanism between general and instrumental values is the institutional make-up of health care systems. The importance items in our study reflect instrumental values in the sense that they are low in a hierarchy of values where lower values contribute to the realisation of higher values. Solidarity and equity are examples of general values; prescribing pharmaceuticals that are covered by health care insurance could be seen as contributing to equity, and is thus an instrumental value.

In general, we believe that further theorising about differences between health care systems in what people find important, might start from the positive relation between importance and experience, from the idea that people compare their experiences with those of others, and from the idea that general and instrumental values might be related trough the institutions of the health care system. Hofstede [24] has identified a number of general dimensions of culture that might also be related to instrumental health care values through different types of welfare states [30].

The analysis presented in this article has its shortcomings. The number of countries is small. The analysis of differences between countries is based on only twelve countries, even though large numbers of respondents were involved. The number respondents increase our confidence in the averages per country, but this does not solve the problem of small numbers at the country level. Differences between user groups could not be taken into account because of the small number of groups for all countries except the Netherlands, although one might expect these differences to exist [29].

The multilevel model now contained two levels, respondents and countries. However, as table 1 indicated, the selection of respondents was through health care institutions. Hence, a level between respondents and countries should ideally have been specified. However, the data did not allow this. Differences between user groups are relatively large. However, the estimated intraclass correlations of table 2 seem to refer more to variation between countries than between user groups. Future research with larger numbers of user groups would be helpful to be able to take case mix differences into account.

We used existing data, collected in different studies with different aims and methods. The response rates differed across studies. The user groups in the surveys refer to are very different, ranging from GP-patients in Belarus to disabled people in the UK and IBD patients in Israel. Apart from the apparent differences between user groups, there are also differences between countries in the position and tasks of GPs. The situation in Ukraine and Belarus was transitional, even ten years after the fall of the Iron Curtain [31]. This may have reduced comparability and create variations in respondents and services to be evaluated.

The QUOTE-questionnaires provide a general framework, and researchers have adapted them to their own aims. Against the advantage of flexible adaptation to different research aims and populations stands the disadvantage of reduced (international) comparability. For (international) comparisons more standardisation is very important. Apart from that, the ten QUOTE-aspects we used in our analyses cover different dimensions of the Quality of care concept and can be understood as being comprehensive enough for this explorative type of research.

In conclusion, we believe it is important to continue to do research into health care related values because of the increasing importance of user views, both in the health policies of European countries separately and in the international debate about the performance of health care systems. There is not much ground for strong cultural relativism, saying that what is important in the eyes of health care users is so different that it is not possible to develop performance measures that can be used in a wide range of countries.

## Conclusion

There are differences between countries in the importance people attach to aspects of health care. Most of this variation is related to individual differences, but there is also significant variation between countries. The ranking of aspects shows similarities between countries. In nearly all countries, people ranked the item that their GP should take them seriously as most important, while an item about waiting time was always ranked lowest. It is difficult to explain the variation between countries. Further theorising should take into account that importance and performance ratings are positively related, that people compare their experiences with those of others, and that general and instrumental values might be related through the institutions of the health care system.

## Competing interests

The author(s) declare that they have no competing interests.

## Authors' contributions

PG conceived the study and wrote the first drafts of the article; JK performed the statistical analysis; HS provided data and wrote the final draft of the article; WB provided data; IvdE provided data.

## Pre-publication history

The pre-publication history for this paper can be accessed here:


